# Fluid balance, intradialytic hypotension, and outcomes in critically ill patients undergoing renal replacement therapy: a cohort study

**DOI:** 10.1186/s13054-014-0624-8

**Published:** 2014-11-18

**Authors:** Jonathan A Silversides, Ruxandra Pinto, Rottem Kuint, Ron Wald, Michelle A Hladunewich, Stephen E Lapinsky, Neill KJ Adhikari

**Affiliations:** Interdepartmental Division of Critical Care, University of Toronto, 30 Bond Street, Toronto, ON M5B 1W8 Canada; Centre for Infection and Immunity, Queens University Belfast, Health Sciences Building, 97 Lisburn Road, Belfast, BT9 7BL UK; Division of Critical Care and Outreach, Belfast Health and Social Care Trust, Belfast City Hospital, Lisburn Road, Belfast, BT9 7AB UK; Programme in Trauma, Emergency, and Critical Care, Sunnybrook Health Sciences Centre, Room D1.08, 2075 Bayview Avenue, Toronto, ON M4N 3M5 Canada; Division of Nephrology, St Michael’s Hospital, 30 Bond Street, Toronto, ON M5B 1W8 Canada; Division of Nephrology, University of Toronto, 190 Elizabeth Street, Suite 3-805, Toronto, ON M5G 2C4 Canada; Division of Nephrology, Sunnybrook Health Sciences Centre, 2075 Bayview Avenue, Room A206a, Toronto, ON M4N 3M5 Canada; Department of Critical Care Medicine, Sunnybrook Health Sciences Centre, Room D1.08, 2075 Bayview Avenue, Toronto, ON M4N 3M5 Canada

## Abstract

**Introduction:**

In this cohort study, we explored the relationship between fluid balance, intradialytic hypotension and outcomes in critically ill patients with acute kidney injury (AKI) who received renal replacement therapy (RRT).

**Methods:**

We analysed prospectively collected registry data on patients older than 16 years who received RRT for at least two days in an intensive care unit at two university-affiliated hospitals. We used multivariable logistic regression to determine the relationship between mean daily fluid balance and intradialytic hypotension, both over seven days following RRT initiation, and the outcomes of hospital mortality and RRT dependence in survivors.

**Results:**

In total, 492 patients were included (299 male (60.8%), mean (standard deviation (SD)) age 62.9 (16.3) years); 251 (51.0%) died in hospital. Independent risk factors for mortality were mean daily fluid balance (odds ratio (OR) 1.36 per 1000 mL positive (95% confidence interval (CI) 1.18 to 1.57), intradialytic hypotension (OR 1.14 per 10% increase in days with intradialytic hypotension (95% CI 1.06 to 1.23)), age (OR 1.15 per five-year increase (95% CI 1.07 to 1.25)), maximum sequential organ failure assessment score on days 1 to 7 (OR 1.21 (95% CI 1.13 to 1.29)), and Charlson comorbidity index (OR 1.28 (95% CI 1.14 to 1.44)); higher baseline creatinine (OR 0.98 per 10 μmol/L (95% CI 0.97 to 0.996)) was associated with lower risk of death. Of 241 hospital survivors, 61 (25.3%) were RRT dependent at discharge. The only independent risk factor for RRT dependence was pre-existing heart failure (OR 3.13 (95% CI 1.46 to 6.74)). Neither mean daily fluid balance nor intradialytic hypotension was associated with RRT dependence in survivors. Associations between these exposures and mortality were similar in sensitivity analyses accounting for immortal time bias and dichotomising mean daily fluid balance as positive or negative. In the subgroup of patients with data on pre-RRT fluid balance, fluid overload at RRT initiation did not modify the association of mean daily fluid balance with mortality.

**Conclusions:**

In this cohort of patients with AKI requiring RRT, a more positive mean daily fluid balance and intradialytic hypotension were associated with hospital mortality but not with RRT dependence at hospital discharge in survivors.

**Electronic supplementary material:**

The online version of this article (doi:10.1186/s13054-014-0624-8) contains supplementary material, which is available to authorized users.

## Introduction

Acute kidney injury (AKI) occurs commonly in critical illness, with 4 to 10% of patients admitted to the intensive care unit (ICU) requiring renal replacement therapy (RRT) [1-3]. In recent studies, the mortality of ICU patients with AKI requiring RRT has been reported to be 40 to 50% [1,4]. Specific therapeutic options for AKI are lacking, and emphasis has traditionally been placed on maintenance of renal perfusion using intravenous fluids, inotropes, and vasopressors. Fluid loading is advocated, particularly in sepsis-related AKI, to treat possible hypovolaemia, improve cardiac output, and maximise renal blood flow [5]. This practice is largely unsupported by evidence and has been challenged by observations that renal blood flow in sepsis may be normal, elevated or decreased [1,4,6], that the renal resistive index, a measure of renal vascular tone, is unchanged in response to fluid challenge in septic patients [5,7], and by numerous observational studies demonstrating an association between fluid overload and mortality in critically ill adults and children with AKI [1-4,8-12]. It is unclear whether this association reflects confounding by greater severity of illness in patients whose intravenous fluid requirements are higher or in whom efforts to remove fluid are poorly tolerated, or whether it simply reflects variation in the timing of RRT initiation. However, variation in the timing of RRT initiation is unlikely to be the sole explanation for the association between fluid overload and mortality, since it has been demonstrated in cohorts that included AKI patients managed without RRT [9,10] and has been noted at time points other than at RRT initiation [10]. It is also unclear whether treatment with diuretics or ultrafiltration is effective in modifying this relationship.

We conducted a cohort study to investigate the relationship between fluid balance and outcome in critically ill patients with AKI requiring RRT. We hypothesised that positive fluid balance and intradialytic hypotension during the first seven days after RRT initiation would be associated with both mortality and RRT dependence at hospital discharge.

## Methods

### Design and setting

This cohort study used data from the Toronto Acute Kidney Injury registry. The registry is a prospectively collected database of patients who started RRT for AKI at two university-affiliated hospitals (April 2007 to October 2012 at St Michael’s Hospital and December 2009 to December 2011 at Sunnybrook Health Sciences Centre, Toronto, ON, Canada) and includes information such as patient demographics, baseline admission characteristics, comorbidities identified in medical records, fluid balance, RRT variables, vital signs during RRT, and outcomes. In both centres, RRT is initiated by the nephrology team in collaboration with the critical care service, according to standard indications; we did not collect data on the specific indication(s) for each patient. Both teams agreed on modality (intermittent hemodialysis or continuous renal replacement therapy, available at both hospitals, or sustained low-efficiency dialysis, available at St Michael’s Hospital only) and fluid management goals.

### Patients

We considered patients aged 16 years or older who received RRT for ≥2 days in an ICU at the two study hospitals (including a coronary care unit at St Michael’s Hospital) during the time period noted above, and for whom fluid balance data were available for ≥1 day of their ICU stay. Patients were excluded from the registry if RRT was initiated to treat a toxic ingestion, if they had pre-existing end-stage renal disease or had received a renal transplant in the year prior to hospital admission.

### Definitions

We defined day 1 as the 24-hour period, or part thereof, on which RRT was initiated. Daily fluid balance was defined as total fluid intake from all sources (intravenous fluids and blood products, enteral and parenteral nutrition, and medications) minus output (urine, ultrafiltrate, output from drains, and gastrointestinal losses). We calculated mean daily fluid balance for the first seven days following RRT initiation, using the number of days with data available if less than seven. Cumulative fluid balance was the total fluid balance between ICU admission and the day of RRT initiation. Fluid overload at RRT commencement was defined as positive cumulative fluid balance >10% of admission weight, as in previous studies [1,4,8,11]. We did not collect data on type of intravenous fluid used (balanced or unbalanced crystalloids or colloids).

One session of RRT was defined as continuous renal replacement therapy (CRRT) for a 24-hour period or part thereof or one session of either intermittent hemodialysis or sustained low-efficiency dialysis (SLED). Baseline creatinine was defined as premorbid creatinine or the first creatinine during the index hospital admission if premorbid creatinine was unavailable. Intradialytic hypotension was defined for each session of RRT as either the occurrence of mean arterial pressure (MAP) <65 mmHg or a reduction of ≥20% from the starting MAP, consistent with widely used definitions of absolute [5,13] and relative [14,15] hypotension, and was expressed as a percentage of days (up to seven) with available data. We lacked data on duration of hypotension and frequency of individual episodes, but all patients were in an ICU or coronary care unit, suggesting that hypotension would be recognised and treated promptly. We did not take into consideration the use of vasopressors to prevent or mitigate intradialytic hypotension because we lacked details of vasopressor dosing during RRT; instead, we determined whether patients received any vasopressor support during the first seven days of RRT.

### Data collection

For eligible patients, we abstracted registry data on primary diagnoses, maximum sequential organ failure assessment (SOFA) scores [6,16] on days 1, 3 and 5 (where day 1 is the first day of RRT), Charlson comorbidity index [7,17], laboratory values, daily fluid balance data on days 1 to 7, vital signs, receipt of a vasopressor, and outcomes at hospital discharge. We reviewed the registry data for missing or incorrect data items, and sought missing data elements from available patient records.

### Statistical analysis and outcomes

Continuous variables were summarized as mean (standard deviation, SD) or median (quartile 1 (Q1), quartile 3 (Q3)) as appropriate, and discrete variables are presented as frequencies and percentages.

We constructed logistic regression models for the outcomes of hospital mortality and RRT dependence at hospital discharge in survivors, with mean daily fluid balance, intradialytic hypotension and sex forced into both models. Other covariates were assessed using univariable logistic regression models; selection criteria for covariate inclusion in the final models were univariable *P* ≤0.2, clinical plausibility, and absence of important multicollinearity (*P* <0.8). We did not consider interaction terms in the absence of clinically compelling reasons to believe that any covariate would modify the effect of the main exposure variables. We did not consider fluid overload at RRT commencement as an independent variable in the main analyses due to the extent of missing data. Results are expressed as odds ratios (ORs) with 95% confidence intervals (CIs). We assessed the relationship between continuous covariates and log odds of the outcomes graphically and detected no departures from the assumption of linearity.

We anticipated that the problem of immortal time bias would lead to an overestimation of the strength of the associations between the exposures of mean daily fluid balance and intradialytic hypotension and the outcome of hospital mortality. This bias arises because a patient who survives longer has more time to develop a negative fluid balance and intradialytic normotension [18,19]. We addressed this possibility in a sensitivity analysis by adjusting for number of days with available fluid balance data available (up to seven); in this analysis, the main exposures were mean daily fluid balance (as in the primary analysis) and days with intradialytic hypotension (up to seven). This bias would not affect our analysis of RRT dependence in hospital survivors.

We conducted two additional sensitivity analyses. First, we analysed the same two outcomes with the mean daily fluid balance dichotomised as positive or negative. Second, to investigate whether fluid overload at commencement of RRT modified the relationship between mean daily fluid balance and mortality, we constructed a *post hoc* logistic regression model in the subgroup of patients with data available to calculate fluid overload. We used the same adjustment variables as in the main model but also adjusted for fluid overload prior to RRT, dichotomised as >10% vs. ≤10%, and the interaction term between mean daily fluid balance on RRT and fluid overload. We did not consider a similar logistic regression model for RRT dependence in hospital survivors in fluid overloaded patients due to few outcome events.

Data were recorded in a Microsoft (Microsoft, Redmond, WA, USA) Access database. All analyses were done using SAS version 9.3 (SAS Institute Inc., Cary, NC, USA.) or R version 2.15.3 (The R Foundation for Statistical Computing, Vienna, Austria). All tests are two-sided; we considered *P* <0.05 to be statistically significant.

### Ethics

The Research Ethics Boards of St Michael’s Hospital and Sunnybrook Health Sciences Centre approved this study. In view of the observational nature of the study and measures to maintain confidentiality of patient data, the requirement for individual patient consent was waived.

## Results

**Figure 1:**
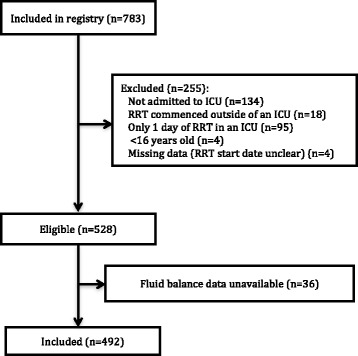
**Flow of patients through the study.**

**Figure 2:**
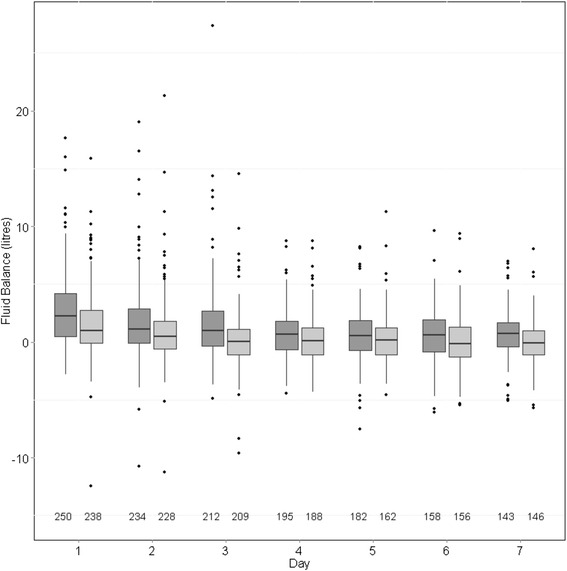
**Fluid balance on each of the first seven days after starting renal replacement therapy (RRT), in decedents at hospital discharge (dark grey) vs. survivors (light grey).** The number of patients with data is below each column. For each column, the dark horizontal line denotes the median value, the bottom and top of the box denote the first and third quartiles respectively, the upper whisker extends to the highest value that is within (1.5 × interquartile range) of the third quartile, and the lower whisker extends to the lowest value within (1.5 × interquartile range) of the first quartile. Data beyond the end of the whiskers are outliers and plotted as points.

**Figure 3:**
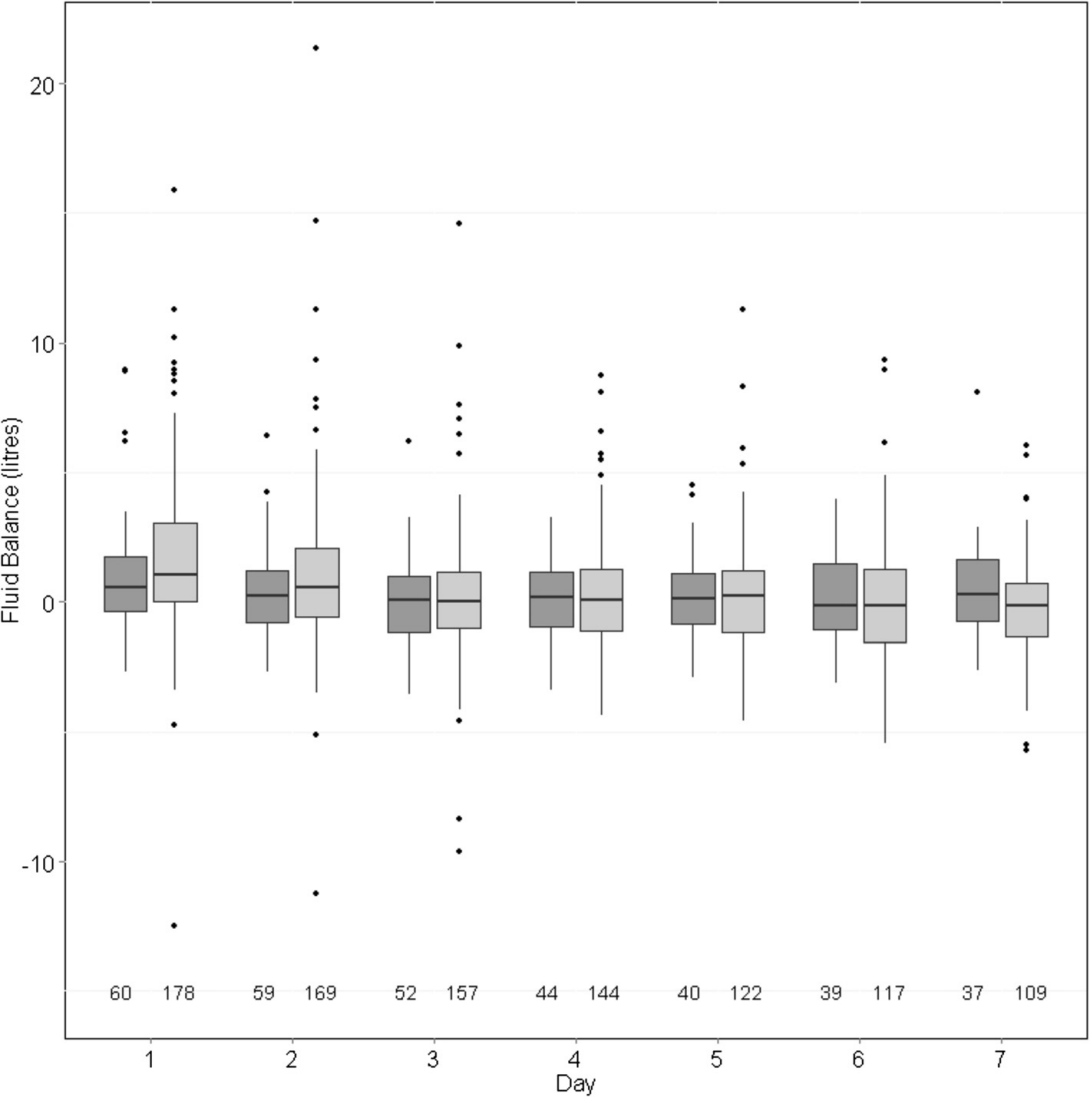
**Fluid balance on each of the first seven days after starting renal replacement therapy (RRT), in survivors to hospital discharge in patients who were RRT dependent (dark grey) vs. RRT free (light grey).** The number of patients with data is below each column. For each column, the dark horizontal line denotes the median value, the bottom and top of the box denote the first and third quartiles, the upper whisker extends to the highest value that is within (1.5 × interquartile range) of the third quartile, and the lower whisker extends to the lowest value within (1.5 × interquartile range) of the first quartile. Data beyond the end of the whiskers are outliers and plotted as points.

A total of 492 patients, of whom 299 (60.8%) were male, met criteria for inclusion in the study (Figure 1). The mean (SD) age was 62.9 (16.3) years, the mean (SD) SOFA score at ICU admission was 12.1 (4.2), and 251 (51.0%) patients died in hospital. Of the 241 survivors, 61 (25.3%) required RRT at hospital discharge. Further patient characteristics are given in Table 1. Intradialytic hypotension was common, occurring in 428 (87.3%) of patients on one or more occasions. The median (Q1, Q3) of mean daily fluid balances over the first seven days of RRT in the entire cohort was 708 (−69, 1805) mL, with a positive mean fluid balance in 353 (71.7%) of patients. Mean daily fluid balance differed between hospital decedents (1134 (242, 2556) mL) and survivors (413 (−371, 1106) mL; *P* <0.01), but not between survivors who were RRT dependent at hospital discharge (71 (−330, 898) mL) and those who were not (475 (−400, 1310) mL; *P* = 0.36). Figures 2 and 3 show daily fluid balance over time in hospital decedents vs. survivors and in survivors requiring RRT vs. free of RRT at hospital discharge, respectively.

**Table 1:** **Characteristics of patients in the cohort**

**Variable**	**Whole cohort**	**Survivors**	**Non-survivors**	***P*** **value**
	**(n = 492)**	**(n = 241)**	**(n = 251)**	
*Demographics*				
Age, years	62.9 (16.3)	61.0 (16.6)	64.7 (15.7)	0.01
Male	299 (60.8%)	148 (61.4%)	151 (60.2%)	0.78
Weight, kg (n = 413, 197, 216)	85.2 (24.3)	86.2 (24.4)	84.3 (24.2)	0.43
*Comorbidities*				
Charlson comorbidity index	2 (1, 3)	2 (1, 3)	2 (1, 4)	<0.001
Congestive heart failure	89 (18.1%)	43 (17.8%)	46 (18.3%)	0.89
Liver cirrhosis	20 (4.1%)	4 (1.7%)	16 (6.4%)	0.01
Baseline creatinine, μmol/L (n = 490, 240, 250)	123.5 (86, 206)	136.5 (92, 223)	110 (83, 190)	<0.001
*Details of ICU admission*				
Admission type				
Medical	241 (49.0%)	115 (47.7%)	126 (50.2%)	0.58
Surgical	251 (51.0%)	126 (52.3%)	125 (49.8%)	
Cardiac surgery	86 (17.5%)	47 (19.5%)	39 (15.5%)	0.25
Aortic aneurysm repair	29 (5.9%)	19 (7.9%)	10 (4.0%)	0.07
Admission diagnosis (n = 485, 238, 247)				<0.001
Trauma	25 (5.2%)	15 (6.3%)	10 (4.1%)	
Cardiovascular	198 (40.8%)	106 (44.5%)	92 (37.3%)	
Respiratory	59 (12.2%)	24 (10.1%)	35 (14.2%)	
Gastrointestinal	91 (18.8%)	26 (10.9%)	65 (26.3%)	
Neurological	11 (2.3%)	6 (2.5%)	5 (2.0%)	
Renal	54 (11.1%)	37 (15.6%)	17 (6.9%)	
Other	47 (9.7%)	24 (10.1%)	23 (9.3%)	
SOFA on day of ICU admission (n = 347, 164, 183)	12.1 (4.2)	11.6 (4.2)	12.5 (4.1)	0.05
SOFA - cardiovascular on day of ICU admission (n = 341, 161, 180)	3 (1, 4)	3 (1, 4)	3 (1, 4)	0.17
Any vasopressor use on days 1-7	272 (55.3%)	100 (41.5%)	172 (68.5%)	<0.001
*Details of RRT*				
SOFA on day RRT commenced (n = 490, 239, 251)	14.2 (4.2)	12.8 (3.9)	15.6 (4.1)	<0.001
SOFA - cardiovascular on day RRT commenced (n = 490, 239, 251)	3 (1, 4)	3 (1, 4)	4 (2, 4)	<0.001
Days from ICU admission to RRT	2 (1, 5.5)	2 (1, 4)	2 (1, 7)	0.002
Initial RRT modality				<0.001
IHD	212 (43.1%)	129 (53.5%)	83 (33.1%)	
SLED	61 (12.4%)	31 (12.9%)	30 (12.0%)	
CRRT	219 (44.5%)	81 (33.6%)	138 (55.0%)	
Days of RRT	7 (4, 13)	7 (4, 14)	6 (3, 12)	0.015
Mean daily fluid balance, mL	708 (−69, 1805)	413 (−371, 1106)	1134 (242, 2556)	<0.001
Positive mean daily fluid balance on days 1-7	353 (71.7%)	151 (62.7%)	202 (80.5%)	<0.001
Intradialytic hypotension on any day (n = 490, 240, 250)	428 (87.3%)	194 (80.8%)	234 (93.6%)	<0.001
Intradialytic hypotension (% days up to 7) (n = 490, 240, 250)	50.0 (28.6, 80.0)	42.9 (20.0, 66.7)	66.7 (42.9, 100)	<0.001
*Clinical outcomes*				
Days of ICU stay	14 (7, 26.5)	14 (7, 29)	14 (8, 24)	0.75
Days of hospital stay	28 (15.5, 48)	34 (23, 58)	21 (11, 39)	<0.001

Dichotomous data are of the form n (%) and continuous data of the form mean (SD) or median (Q1, Q3). The number of patients with data is provided where this differs from the total. *P* values are based on univariable logistic regression, except for days of ICU and hospital stay, days between ICU admission and RRT, and days of RRT, for which Wilcoxon signed-rank tests are reported. CRRT, continuous renal replacement therapy; ICU, intensive care unit; IHD, intermittent hemodialysis; RRT, renal replacement therapy; SLED, sustained low-efficiency dialysis; SOFA, sequential organ failure assessment score.

### Hospital mortality

Our main exposures of mean daily fluid balance and intradialytic hypotension were only weakly correlated (Spearman’s correlation 0.23). In our final multivariable logistic regression model (Table 2), independent risk factors for mortality included mean daily fluid balance (OR 1.36 per 1000 mL more positive, 95% CI 1.18 to 1.57), intradialytic hypotension (OR 1.14 per 10% increase in number of days with intradialytic hypotension, 95% CI 1.06 to 1.23), higher age (OR 1.15 per 5 years, 95% CI 1.07 to 1.25), higher Charlson comorbidity index (OR 1.28, 95% CI 1.14 to 1.44), and higher maximum SOFA score (OR 1.21, 95% CI 1.13 to 1.29) on days 1 to 7 of RRT were associated with increased mortality, while higher baseline creatinine (OR 0.98 per 10 μmol/l, 95% CI 0.97 to 0.996) was associated with lower mortality. These findings were similar in a sensitivity analysis adjusting for number of days with fluid balance available (OR per 1000 mL mean daily fluid balance 1.41, 95% CI 1.22 to 1.63; OR per 10% increase in days of intradialytic hypotension 1.16, 95% CI 1.02 to 1.32). Multiple logistic regression with fluid balance dichotomised gave similar results: adjusted OR for positive vs. negative mean daily fluid balance 1.72, 95% CI 1.06 to 2.81.

**Table 2:** **Final multiple logistic regression model for mortality**

**Variable**	**Unadjusted OR**	***P*** **value**	**Adjusted OR**	***P*** **value**
	**(95% CI)**		**(95% CI)**	
Mean daily fluid balance (per 1000 mL positive)	1.47 (1.30-1.66)	<0.001	1.36 (1.18-1.57)	<0.001
Intradialytic hypotension (per 10% increase in number of days)	1.27 (1.19-1.35)	<0.001	1.14 (1.06-1.23)	<0.001
Age (per 5-year increase)	1.07 (1.02-1.14)	0.01	1.15 (1.07-1.25)	<0.001
Male sex	0.95 (0.66-1.36)	0.78	1.04 (0.66-1.64)	0.85
Charlson comorbidity index	1.16 (1.07-1.26)	<0.001	1.28 (1.14-1.44)	<0.001
Primary renal diagnosis	0.44 (0.24-0.78)	0.005	0.82 (0.40-1.66)	0.58
Baseline creatinine (per 10 μmol/l increase)	0.98 (0.97-0.99)	<0.001	0.98 (0.97-0.996)	0.01
Abdominal aortic aneurysm repair	0.48 (0.22-1.07)	0.07	0.52 (0.20-1.36)	0.18
Maximum SOFA on days 1-7	1.22 (1.16-1.28)	<0.001	1.21 (1.13-1.29)	<0.001
Any vasopressors on days 1-7	3.07 (2.12-4.44)	<0.001	1.58 (0.96-2.62)	0.07
Initial RRT modality				
CRRT vs. IHD	2.65 (1.79-3.91)	<0.001	0.59 (0.34-1.02)	0.06
SLED vs. IHD	1.50 (0.85-2.67)	0.16	0.69 (0.34-1.40)	0.30

The final model had 492 patients and 251 deaths; it did not demonstrate lack of goodness of fit (Hosmer-Lemeshow *P* = 0.85). CI, confidence interval; CRRT, continuous renal replacement therapy; IHD, intermittent hemodialysis; OR, odds ratio; RRT, renal replacement therapy; SLED, sustained low-efficiency dialysis; SOFA, sequential organ failure assessment score.

### RRT dependence in hospital survivors

Univariable comparisons are given in Additional file 1. Our main exposures of mean daily fluid balance and intradialytic hypotension were not strongly correlated (Spearman’s correlation 0.06). In our final model (Table 3), neither mean daily fluid balance (OR 0.93 per 1000 mL, 95% CI 0.75 to 1.16) nor intradialytic hypotension (OR 1.02 per 10% increase in number of days with intradialytic hypotension, 95% CI 0.92 to 1.13) were associated with RRT dependence at hospital discharge; the only independent predictor was a pre-existing diagnosis of congestive heart failure (OR 3.13, 95% CI 1.46 to 6.74). A sensitivity analysis using multiple logistic regression with fluid balance dichotomised gave similar results: adjusted OR for positive vs. negative mean daily fluid balance 0.72, 95% CI 0.38 to 1.35.

**Table 3:** **Final multiple logistic regression model for RRT dependence at hospital discharge in survivors.**

**Variable**	**Unadjusted OR**	***P*** **value**	**Adjusted OR**	***P*** **value**
	**(95% CI)**		**(95% CI)**	
Mean daily fluid balance (per 1000 mL positive)	0.91 (0.74-1.12)	0.36	0.93 (0.75-1.16)	0.54
Intradialytic hypotension (per 10% increase in number of days)	1.01 (0.92-1.11)	0.86	1.02 (0.92-1.13)	0.69
Male sex	1.05 (0.58-1.91)	0.87	1.20 (0.62-2.31)	0.59
Congestive heart failure	2.60 (1.30-5.19)	0.007	3.13 (1.46-6.74)	0.003
Medical (versus surgical) diagnosis	1.84 (1.02-3.32)	0.04	1.75 (0.88-3.47)	0.11
Primary renal diagnosis	2.02 (0.97-4.23)	0.06	1.72 (0.75-3.94)	0.20
Baseline creatinine (per 10 μmol/l increase)	1.01 (0.999-1.02)	0.07	1.01 (0.995-1.02)	0.23
Maximum SOFA on days 1-7	0.94 (0.87-1.01)	0.10	0.98 (0.89-1.07)	0.65
Initial RRT modality				
CRRT vs. IHD	0.52 (0.26-1.04)	0.06	0.72 (0.31-1.65)	0.43
SLED vs. IHD	1.18 (0.51-2.75)	0.70	1.51 (0.60-3.83)	0.38

The final model had 241 survivors and 61 patients with RRT dependence at hospital discharge; it did not demonstrate lack of goodness of fit (Hosmer-Lemeshow *P* = 0.69). CI, confidence interval; CRRT, continuous renal replacement therapy; IHD, intermittent hemodialysis; OR, odds ratio; RRT, renal replacement therapy; SLED, sustained low-efficiency dialysis; SOFA, sequential organ failure assessment score.

### Interaction of fluid overload at RRT commencement and mean daily fluid balance on RRT

Three hundred and eight (62.6%) patients in the cohort had sufficient data to calculate fluid overload at the start of RRT; of those, 125 (40.6%) had >10% fluid overload. Compared to patients without fluid overload, those with >10% fluid overload (Additional file 2) weighed less on admission, had lower baseline creatinine, had different admission diagnoses, started RRT later in the ICU admission and with more organ dysfunction, and were more likely to die in hospital. In this subgroup of patients for whom fluid overload was calculable, >10% fluid overload at commencement of RRT was associated with mortality (adjusted OR 2.03, 95% CI 1.12 to 3.68), but only weakly correlated with mean daily fluid balance (Spearman’s coefficient 0.03). There was no interaction between the effects of fluid overload and mean daily fluid balance (interaction *P* = 0.72). The associations between mortality and the main exposures of mean daily fluid balance (adjusted OR 1.53 per 1000 mL, 95% CI 1.25 to 1.87) and intradialytic hypotension (1.18 per 10% increase in number of days with intradialytic hypotension, 95% CI 1.06 to 1.31) remained similar to the original model (Table 4).

**Table 4:** **Final multiple logistic regression model for mortality in subgroup of patients with cumulative fluid balance and weight available**

**Variable**	**Unadjusted OR**	***P*** **value**	**Adjusted OR**	***P*** **value**
	**(95% CI)**		**(95% CI)**	
Mean daily fluid balance (per 1000 mL positive)	1.67 (1.40-1.99)	<0.001	1.53 (1.25-1.87)	<0.001
Intradialytic hypotension (per 10% increase in number of days)	1.31 (1.21-1.43)	<0.001	1.18 (1.06-1.31)	0.002
Fluid overload >10% vs. ≤10%	2.27 (1.42-3.64)	0.006	2.03 (1.12-3.68)	0.02
Age (per 5-year increase)	1.04 (0.98-1.12)	0.21	1.11 (1.01-1.22)	0.02
Male sex	0.94 (0.59-1.48)	0.78	1.14 (0.64-2.04)	0.66
Charlson comorbidity index	1.09 (0.98-1.21)	0.10	1.21 (1.05-1.39)	0.01
Abdominal aortic aneurysm repair	0.55 (0.23-1.34)	0.19	0.65 (0.22, 1.96)	0.44
Primary renal diagnosis	0.55 (0.27-1.13)	0.10	1.31 (0.53-3.26)	0.56
Baseline creatinine (per 10 μmol/l increase)	0.97 (0.96-0.99)	0.003	0.99 (0.97-1.01)	0.24
Maximum SOFA on days 1-7	1.24 (1.16-1.32)	<0.001	1.15 (1.05-1.26)	0.002
Any vasopressors on days 1-7	3.37 (2.07-5.46)	<0.001	1.52 (0.78-2.95)	0.22
Initial RRT modality				
CRRT vs. IHD	3.11 (1.89, 5.11)	<0.001	0.77 (0.39-1.52)	0.45
SLED vs. IHD	1.18 (0.56, 2.51)	0.66	0.51 (0.20-1.32)	0.16

The final model had 308 patients and 168 deaths; it did not demonstrate lack of goodness of fit (Hosmer Lemeshow *P* = 0.62). There was no evidence of interaction between mean daily fluid balance and fluid overload (*P* = 0.72); we therefore report the results of the model with no interaction term. CI, confidence interval; CRRT, continuous renal replacement therapy; IHD, intermittent hemodialysis; OR, odds ratio; RRT, renal replacement therapy; SLED, sustained low-efficiency dialysis; SOFA, sequential organ failure assessment score.

## Discussion

In this large and heterogeneous cohort of critically ill patients with AKI who received RRT, a more positive mean daily fluid balance and intradialytic hypotension in the week after RRT initiation were independently associated with an increased risk of hospital mortality, but not RRT dependence at hospital discharge in survivors. Fluid overload at commencement of RRT was associated with mortality in this cohort, as in previous studies [4,8-10]. However, our main focus was on mean daily fluid balance in the week after RRT initiation, taking into account the risk of intradialytic hypotension with fluid removal, which we believed to be a clinically relevant variable with important therapeutic implications. The initiation of RRT brings with it the opportunity for clinicians to proactively and rigorously control fluid balance with relative ease, and thus the influence of fluid management on clinical outcomes has important implications for both the timing and goals of RRT.

Biological mechanisms have been proposed linking fluid overload to disruption of the endothelial glycocalyx, interstitial edema, multiple organ damage, and death [20,21]. Limited support for the injurious effects of fluid overload on renal and other organ function comes from secondary analyses of two large randomised trials. One study found a negative mean daily fluid balance to be associated with lower 90-day mortality and increased RRT-free days in a large trial of high- vs. low-dose RRT for AKI [4], and another found a lower incidence of AKI in patients with acute respiratory distress syndrome randomized to a restrictive vs. liberal fluid management group, after correction for the dilutional effect of a positive fluid balance [22]. These associations between less fluid accumulation and improved outcomes suggest that clinicians may consider ultrafiltration to minimise or remove accumulated fluid once patients commence RRT.

We hypothesised that limiting or removing fluid overload after patients commenced RRT for AKI may ameliorate AKI severity and improve renal recovery in survivors. Although we found that a more positive fluid balance on RRT was associated with mortality, there was no association with RRT dependence at hospital discharge. Unsurprisingly, we found that intradialytic hypotension was associated with mortality. However, despite evidence that relative hypotension is associated with AKI onset in a non-critically ill hospital population [23], we found no association between intradialytic hypotension and RRT dependence. Although this finding may arise from lack of statistical power, another possible interpretation is that mild hypotension in the course of fluid removal may not reduce the probability of renal recovery in patients who survive.

Our study adds to current understanding of the relationship between fluid balance and outcomes. While previous studies have demonstrated an association between fluid overload at selected time points and death, ours is only the second study after that of Bellomo *et al*. [4] to demonstrate the relationship between positive fluid balance on RRT and mortality in critically ill adults, and suggests that fluid management during RRT is an important target for future clinical trials. Our sensitivity analysis adjusting for fluid accumulation at RRT initiation suggests that this relationship is not confined to those with measured fluid overload. Our cohort comprises a heterogeneous and unselected sample of critically ill patients for whom clinicians made the decision to start RRT, and demonstrates the safety of a less positive fluid management strategy.

The main limitation of this and previous similar studies is that risk adjustment cannot eliminate the possibility of residual confounding. While we adjusted for confounders such as severity of physiological derangement (SOFA score), premorbid health (Charlson comorbidity index), cardiovascular instability (use of vasopressors and intradialytic hypotension), and age, we lacked data on fluid overload at the time of RRT initiation in 40% of the cohort and therefore could not adjust for this covariate without a substantial loss of statistical power. However, sensitivity analysis in patients in whom fluid overload was calculable produced similar results to that in the whole cohort. It is also possible that other unknown confounders influenced both fluid requirements and the outcomes of mortality and RRT dependence in survivors. Immortal time bias was also a potential problem; however, a sensitivity analysis that accounted for the number of days with data available did not materially alter our findings. Residual confounding may have contributed to the surprising findings that higher baseline creatinine was associated with lower risk of death, but not with RRT dependence at discharge. These associations may reflect the occurrence of RRT-dependent AKI with less severe systemic illness among patients with pre-existing chronic kidney disease.

Other important limitations relating to the observational nature of this study include the lack of standardised criteria for the initiation of and management of RRT, leading to fluid overload as the primary indication for RRT in some patients, the lack of data on the relative contribution of intake and output to overall fluid balance, and missing data. Other limitations of data restricted the calculation of fluid balance to the first seven days following RRT initiation. Although the endpoints of mortality and RRT dependence at hospital discharge are clinically important, some patients may have been transferred to other acute care hospitals, with less certainly regarding final disposition. We defined intradialytic hypotension to include both absolute and relative hypotension and considered the frequency as a percentage of the total number of RRT sessions during which it occurred. However, similar to other AKI studies that reported details of hypotension [23], we lacked data on the duration of each episode and the frequency within individual RRT sessions. Finally, our study included data from only two hospitals, limiting applicability to other centres.

## Conclusions

In summary, our study provides supportive evidence for reduced mortality with a less positive fluid balance after critically ill patients commence RRT. A less positive fluid management strategy does not appear to influence RRT dependence at hospital discharge. Further multicentre cohort investigations, if confirming these findings, would support the design of a randomized trial of an aggressive vs. less aggressive approach to fluid removal in this population.

## Key messages

- A more positive fluid balance and intradialytic hypotension are associated with increased risk of death in a cohort of critically ill patients undergoing renal replacement therapy
- Fluid balance and intradialytic hypotension are not associated with requirement for renal replacement therapy at the time of hospital discharge
- Clinicians may consider using ultrafiltration to minimise fluid overload in critically ill patients with acute kidney injury receiving renal replacement therapy

## Additional files

Additional file 1
**Distribution of risk factors and outcomes for renal replacement therapy (RRT) dependence at hospital discharge in survivors.** Description: This table shows the distribution of risk factors, with *P* values, between groups of hospital survivors after commencement of RRT who were dependent on RRT vs. not at time of discharge. Additional file 2
**Patient characteristics and outcomes for subgroups with missing data, and those with and without >10% fluid overload at renal replacement therapy (RRT) initiation.**

## Abbreviations

AKI: acute kidney injury
CI: confidence interval
CRRT: continuous renal replacement therapy
ICU: intensive care unit
IHD: intermittent hemodialysis
MAP: mean arterial pressure
OR: odds ratio
RRT: renal replacement therapy
SD: standard deviation
SLED: sustained low-efficiency dialysis
SOFA: sequential organ failure assessment
