# Supplementary material for: Fluid balance, intradialytic hypotension, and outcomes in critically ill patients undergoing renal replacement therapy: a cohort study
Source: Crit Care. 2014 Nov 18;18(6):624. doi: 10.1186/s13054-014-0624-8 (PMC4255668; doi:10.1186/s13054-014-0624-8)
Supplement: Additional file 1 — Distribution of risk factors and outcomes for renal replacement therapy (RRT) dependence at hospital discharge in survivors. Description: This table shows the distribution of risk factors, with P values, between groups of hospital survivors after commencement of RRT who were dependent on RRT vs. not at time of discharge. [file 13054_2014_624_MOESM1_ESM.pdf]

Appendix 1. Distribution of risk factors and outcomes for RRT dependence at hospital discharge in survivors

Variable	All Survivors (n=241)	RRT free at discharge (n=180)	RRT-dependent at discharge (n=61)	p-value
Demographics				
Age, years	61.0 (16.6)	61.1 (16.7)	60.5 (16.6)	0.82
Male	148 (61.4%)	110 (61.1%)	38 (62.3%)	0.87
Weight, kg (n=197, 147, 50)	86.2 (24.4)	87.4 (25.4)	82.4 (21.2)	0.22
Comorbidities				
Charlson comorbidity index	2 (1, 3)	2 (0,3)	1 (1,3)	0.54
Congestive heart failure	43 (17.8%)	25 (13.9)	18 (29.5)	<0.01
Liver cirrhosis	4 (1.7%)	4 (2.2)	0 (0)	0.99
Baseline creatinine, μmol/L (n=491, 179, 61)	136.6 (86, 206)	125 (89, 198)	178 (109, 305)	0.07
Details of ICU Admission				
Admission Type				
Medical	115 (47.7%)	79 (43.9%)	36 (59.0%)	0.04
Surgical	126 (52.3%)	101 (56.1%)	25 (41.0%)	
Cardiac surgery	47 (19.5%)	37 (20.6%)	10 (16.4%)	0.48
Aortic aneurysm repair	19 (7.9%)	14 (7.8%)	5 (8.2%)	0.92
Admission Diagnosis (n=238)				
Trauma	15 (6.3%)	13 (7.3%)	2 (3.4%)	0.13
Cardiovascular	106 (44.5%)	78 (43.6%)	28 (47.5%)	
Respiratory	24 (10.1%)	21 (11.7%)	3 (5.1%)	
Gastrointestinal	26 (10.9%)	20 (11.2%)	6 (10.2%)	
Neurological	6 (2.5%)	3 (1.7%)	3 (5.1%)	
Renal	37 (15.6%)	23 (12.9%)	14 (23.7%)	
Other	24 (10.1%)	21 (11.7%)	3 (5.1%)	
SOFA on day of ICU admission (n=164, 124, 40)	11.6 (4.2)	11.7 (4.2)	11.3 (4.2)	0.59
SOFA-cardiovascular on day of ICU admission (n=161, 121, 40)	3 (1,4)	3 (1, 4)	2 (1, 3.5)	0.07
Any vasopressor use on days 1-7	100 (41.5%)	76 (42.2%)	24 (39.3%)	0.69
Details of RRT				
SOFA on day RRT commenced (n=239, 179, 60)	12.8 (3.9)	13.1 (3.9)	12.1 (3.8)	0.08
SOFA-cardiovascular on day RRT commenced (n=239, 179, 60)	3 (1,4)	3 (1, 4)	3 (1, 3.5)	0.67
Days from ICU admission to RRT	2 (1, 4)	2 (1, 4)	1 (0, 3)	0.06
Initial RRT modality				
IHD	129 (53.5%)	92 (51.1%)	37 (60.7%)	0.12
SLED	31 (12.9%)	67 (37.2%)	14 (23.0%)	
CRRT	81 (33.6%)	21 (11.7%)	10 (16.4%)	
Mean daily fluid balance, mL	413 (-371, 1106)	475 (-400, 1310)	71 (-330, 898)	0.36
Positive mean daily fluid balance	151 (62.7%)	116 (64.4%)	35 (57.4%)	0.32
Intradialytic hypotension (% days up to 7) (n=240, 179, 61)	43 (20, 67)	50 (20, 67)	33 (20, 71)	0.86
Days of RRT	7 (4, 14)	6 (4, 12)	14 (8, 23)	<0.001
Clinical outcomes				
Days of ICU stay	14 (7, 29)	14 (7, 28)	12.5 (6, 33)	0.79
Days of hospital stay	34 (23, 58)	33 (20.5, 60)	39 (25, 54)	0.41

Dichotomous data are of the form n (%) and continuous data of the form mean (SD) or median (Q1, Q3). The number of patients with data is provided where this differs from the total. P-values are based on univariable logistic regression, except for days of ICU and hospital stay, days between ICU admission and RRT, and days of RRT, for which Wilcoxon signed-rank tests are reported. Abbreviations: CRRT, continuous renal replacement therapy; ICU, intensive care unit; IHD, intermittent haemodialysis; RRT, renal replacement therapy; SLED, sustained low-efficiency dialysis; SOFA, sequential organ failure assessment score.
