# Supplementary material for: Fluid balance, intradialytic hypotension, and outcomes in critically ill patients undergoing renal replacement therapy: a cohort study
Source: Crit Care. 2014 Nov 18;18(6):624. doi: 10.1186/s13054-014-0624-8 (PMC4255668; doi:10.1186/s13054-014-0624-8)
Supplement: Additional file 2 — Patient characteristics and outcomes for subgroups with missing data, and those with and without >10% fluid overload at renal replacement therapy (RRT) initiation. [file 13054_2014_624_MOESM2_ESM.pdf]

Appendix 2: Patient characteristics and outcomes for subgroups with missing data, and those with and without >10% fluid overload at RRT initiation.

Variable	Whole cohort (n=492)	Missing data (n=182)	>10% fluid overload (n=125)	≤10% fluid overload (n=185)	p-value
Demographics					
Age, years	62.9 (16.2)	64.2 (15.9)	60.9 (16.83)	62.9 (16.2)	0.29
Male	299 (60.8%)	117 (64.3%)	77 (61.6%)	105 (56.8%)	0.40
Weight, kg (n=413)	85.2 (24.3)	87.2 (22.2)	80.7 (19.9)	87.0 (27.6)	0.03
Comorbidities					
Charlson comorbidity index	2 (1, 3)	2 (1, 3)	2 (0, 3)	2 (1, 4)	0.15
Congestive heart failure	89 (18.1%)	36 (19.8)	17 (13.6)	36 (19.5)	0.18
Liver cirrhosis	20 (4.1%)	8 (4.4)	6 (4.8)	6 (3.2)	0.49
Baseline creatinine (n=490, 240, 250)	123.5 (86, 206)	133 (89, 233)	101 (80, 149)	138 (91.5, 222)	<0.01
Details of ICU Admission					
Admission Type					
Medical	241 (49.0%)	111 (61.0%)	46 (36.8%)	84 (45.4%)	0.13
Surgical	251 (51.0%)	71 (39.1%)	79 (63.2%)	101 (54.6%)	
Cardiac surgery	86 (17.5%)	24 (13.2%)	23 (18.4%)	39 (21.1%)	0.56
Aortic aneurysm repair	29 (5.9%)	7 (3.9%)	8 (6.4%)	14 (7.6%)	0.69
Admission Diagnosis (n=485,175,124,184)					
Trauma	25 (5.2%)	12 (6.8%)	7 (5.7%)	6 (3.3%)	<0.001
Cardiovascular	198 (40.8%)	78 (44.1%)	37 (28.2%)	83 (45.1%)	
Respiratory	59 (12.2%)	17 (9.6%)	23 (18.6%)	19 (10.3%)	
Gastrointestinal	91 (18.8%)	29 (16.4%)	35 (28.2%)	27 (14.7%)	
Neurological	11 (2.3%)	5 (2.8%)	2 (1.6%)	4 (2.2%)	
Renal	54 (11.1%)	22 (12.4%)	4 (4.0%)	27 (14.7%)	
Other	47 (9.7%)	14 (7.9%)	15 (12.1%)	18 (9.8%)	
SOFA on day of ICU admission (n=347, 142, 70, 135)	12.1 (4.1)	12.1 (4.2)	12.2 (3.9)	12.05 (4.3)	0.79
SOFA-cardiovascular on day of ICU admission (n=341, 141, 70,130)	3 (1, 4)	3 (1, 4)	4 (1, 4)	3 (1, 4)	0.38
Details of RRT					
SOFA on day RRT commenced (n=490, 181, 125,184	14.2 (4.2)	13.1 (4.4)	15.8 (3.8)	14.3 (4.0)	<0.01
SOFA-cardiovascular on day RRT commenced (n=490, 181,125,184	3 (1, 4)	3 (1, 4)	4 (1, 4)	3 (1, 4)	0.38
Vasopressor use on days 1-7	272 (55.3%)	74 (40.7%)	89 (71.2%)	109 (58.9%)	0.027
Days from ICU admission to RRT	2 (1, 5.5)	1 (0, 5)	4 (2, 8)	2 (1, 4)	<0.01
Initial RRT modality					
IHD	212 (43.1%)	93 (51.1%)	40 (32%)	79 (42.7%)	0.13
SLED	61 (12.4%)	25 (13.7%)	14 (11.2%)	22 (11.9%)	
CRRT	219 (44.5%)	64 (35.2%)	71 (56.8%)	84 (45.4%)	
Days of RRT	7 (4, 13)	7 (4, 13)	7 (4, 14%)	6 (3, 12)	0.27
Mean daily fluid balance, mL	708 (-69, 1805)	657 (-14, 1664)	839 (-166, 2241)	651 (-71.5, 1688)	0.61
Positive mean daily fluid balance	353 (71.7%)	132 (72.5%)	89 (71.2%)	132 (71.3%)	0.98
Intradialytic hypotension (percent of days up to 7), (n=490,180,125, 185)	50.0 (28.6, 80.0)	50 (25, 80)	60 (43, 87)	50 (25, 75)	0.003
Clinical outcomes					
Death	251 (51.0%)	82 (45.1%)	83 (66.4%)	86 (46.5%)	<0.01
RRT dependence at discharge for survivors	61 (25.3.4%)	29 (29%)	8 (19.0%)	24 (24.2%)	0.50

(n=241,100,99,42)					
Days of ICU stay	14 (7, 26.5)	13.5 (7, 28)	18 (9, 28)	13 (6, 24)	<0.01
Days of hospital stay	28 (15.5, 48)	28 (16, 46)	27 (15, 51)	28 (16, 49)	0.62

Dichotomous data are of the form n (%) and continuous data of the form mean (SD) or median (Q1, Q3). The number of patients with data is provided where this differs from the total. All p-values are for comparing the group with fluid overload >10% versus ≤10% and are based on t-test or Wilcoxon rank-sum test as appropriate. Fluid overload at RRT commencement was defined as positive cumulative fluid balance >10% of admission weight. Abbreviations: CRRT, continuous renal replacement therapy; ICU, intensive care unit; IHD, intermittent haemodialysis; RRT, renal replacement therapy; SLED, sustained low-efficiency dialysis; SOFA, sequential organ failure assessment score.
